# 

**Published:** 1886-02

**Authors:** 


CONTENTS.

VOL. 33 No. 2

| | PAGE. | | PAGE. |
|---|---|---|---|
| The Rag Weed | 33 | An Argument for Temperance | 53 |
| Vital Statistics | 35 | Diseases that Kill the Most People | 54 |
| Winter Clothing | 36 | For Melancholia | 56 |
| Never Surrender | 37 | The Poison in Cheese | 56 |
| Pre-Natal Education | 38 | Tea as a Beverage | 58 |
| What Will Cure a Cough | 43 | Painless Tooth Extraction | 58 |
| Bitten by a Centipede | 44 | Beer | 56 |
| A Rapidly Growing Evil | 45 | Botanical Items | 59 |
| A Dangerous Diet | 45 | Will Small Pox Protect Vaccina | 60 |
| Unparalleled Fecundity | 46 | Treatment of Frozen Persons | 61 |
| Milk in Gout | 46 | Caulocorea | 61 |
| Sciatica Relieved by Injection | 47 | Home Department | 62 |
| Reform in the Lunacy Laws | 48 | Recent Publications | 64 |

